# The development of T-cell malignancies in patients with pre-existing myeloproliferative neoplasms: a report of three cases

**DOI:** 10.3332/ecancer.2020.1011

**Published:** 2020-02-17

**Authors:** Ethan A Burns, Kartik Anand, Betty Chung, Shilpan Shah, Jasleen K Randhawa, Sai Ravi Pingali

**Affiliations:** 1 Department of Internal Medicine, Houston Methodist Hospital, 6550 Fannin, Smith Tower, Ste 1101, Houston, TX 77030, USA; 2 Houston Methodist Cancer Center, 6445 Main Street, Outpatient Center, 24th Floor, Houston, TX 77030, USA; 3 Houston Methodist Hospital, Department of Pathology and Genomic Medicine, 6550 Fannin St, Houston, TX 77030, USA; * Equal contribution.

**Keywords:** essential thrombocythemia, primary myelofibrosis, polycythemia vera, Philadelphia negative myeloproliferative neoplasm, angioimmunoblastic T-cell lymphoma, T-cell acute lymphoblastic leukaemia

## Abstract

Secondary acute myeloid leukaemia complicating the natural disease course of pre-existing Philadelphia chromosome-negative myeloproliferative neoplasms (PN-MPN) is well documented and associated with treatment challenges and significant morbidity. The incidence of a T-cell malignancy developing in patients with pre-existing PN-MPN is uncommon, with one case documented in the literature. We present two cases of angioimmunoblastic T-cell lymphoma (AITL) and one case of T-cell acute lymphoblastic leukaemia (T-ALL) that developed in patients with essential thrombocythemia (ET) and primary myelofibrosis (PMF), respectively. All malignancies were advanced at diagnosis and exhibited disease progression, regardless of the mutational status of the underlying ET/PMF, presence of cytogenetic abnormalities, type of T-cell neoplasm or systemic chemotherapy utilised. The median time to diagnosis of AITL or T-ALL from the onset of MPN was 4.5 years (range: 6 months–10 years). This single institutional case series demonstrates the possibility of an association between T-cell neoplasms and PN-MPNs.

## Introduction

Myeloproliferative neoplasms (MPNs) are chronic, clonal diseases defined by the overproduction of fully differentiated cells of haematopoietic origin. In 2016, the World Health Organization Revised Classification System for Haematopoietic Tumours recognised a subcategory of Philadelphia chromosome (PN)-MPN, including polycythemia vera (PV), essential thrombocythemia (ET), primary myelofibrosis (PMF) and prefibrotic PMF [1, 2]. PN-MPNs are phenotypically driven by somatic mutations which culminate in the persistent downstream hyperactivation of the Janus Kinase (JAK)-STAT (signal transducer and activator of transcription) signal transduction pathway. The most common mutations include *JAK2 V617F*, calreticulin (*CALR*) or the thrombopoietin receptor (*MPL*) [3]. Wild type (triple-negative) PMF or ET is not uncommon.

While there are similarities in the clinical, laboratory, morphologic and mutational manifestations of PN-MPNs, variations in survival and progression to leukaemic transformation necessitate an accurate diagnosis [1, 4]. The risk for leukaemic transformation in the decade following the diagnosis of a PN-MPN is 1.3% in ET [5], 2.3% in PV [6] and 10%–20% in PMF [7–9]. The lifetime cumulative incidence of a leukaemic transformation from MPN is approximately 3.8% for ET, 6.8% for PV and 14.2% for PMF [10]. While the most common haematologic malignancies following leukaemic transformation are of myeloid origin, lymphoid malignancies are seldom encountered [4]. The following three cases describe the development of T-cell neoplasms in patients with PN-MPN, a rarely reported occurrence.

## Cases

### Patient 1

A 78-year-old Caucasian woman with a history of pulmonary embolism and triple-negative ET diagnosed 6 months prior and treated with hydroxyurea presented to the hospital with shortness of breath and constipation.

**Figure 1.**
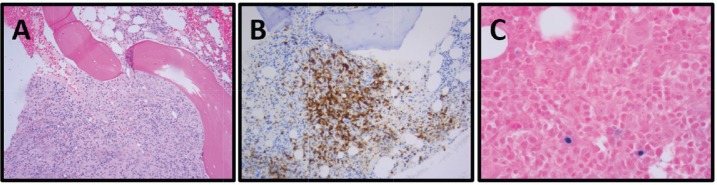
Patient 1, bone marrow biopsy with AITL: (A) Focal, interstitial marrow involvement with monomorphic population of small to medium-sized lymphoid cells with moderately abundant pale eosinophilic cytoplasm, H&E, 100×. (B) PD-1, 100×. (C) EBER ISH, 400×.

**Figure 2.**
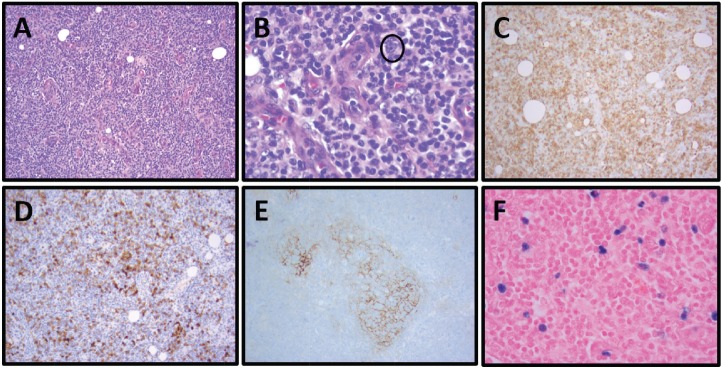
Patient 1, axillary lymph node with AITL: (A) Prominent vascular proliferation of high endothelial venules (HEVs) and nodal architecture effacement by a monomorphic lymphoid population, H&E, 100×. (B) Neoplastic population of follicular helper T cells with small to medium-sized nuclei and clear-to-pale cytoplasm, especially clustering adjacent to HEVs, and scattered CD30+ immunoblasts (circled, IHC not shown). H&E, 400×. (C) Increased numbers of T cells in effaced areas, especially perivascular T cells, CD3, 100x. (D) Increased numbers of neoplastic follicular helper T cells, PD-1, 100×. (E) Expanded dendritic meshwork, CD21, 100×. (F) EBER in situ hybridisation (EBER ISH) positive neoplastic T cells, 100×.

She reported ‘B’ symptoms first noticed 1 month prior to admission. Physical examination was significant for axillary lymphadenopathy (LAD). Significant laboratory findings included hypercalcaemia of 13.6 mg/dL, acute kidney injury with admission creatinine of 2.03 mg/dL and pancytopenia. Computed Tomography (CT) showed splenomegaly (17 cm), diffuse lymphadenopathy and Positron Emission Tomography (PET) demonstrated increased uptake in the splenic and porta hepatis area. Her bone marrow biopsy found atypical lymphoid aggregates that contained a mixed population of T-cells positive for CD3, CD5 and PD1, and B-cells positive for CD20 and variably positive for PAX-5 and BCL6 (Figure 1; Table 1). Epstein Barr Virus encoded small ribonucleic acid (EBER) was highlighted in scattered, predominantly small lymphocytes. Karyotype analysis demonstrated multiple cytogenetic anomalies, with karyotype showing 47,XX,add(2)(p2),add(10)(p11.2),+add(20)(p12)[cp4]/46,XX [16] (Table 1). Excisional axillary lymph node biopsy had immunostaining positive for CD2, CD3, CD5, CD7 and CD4 PD-1, and negative for CD30 in the lesional T-cells, consistent with AITL (Figure 2; Table 1).

She received three cycles of Cyclophosphamide, Doxorubicin Hydrochloride, Etoposide, Vincristine and Prednisone (CHEOP), but repeat PET/CT indicated disease progression in the spleen and increased uptake in her cervical, supraclavicular, axillary, hilar, mediastinal, inguinal and iliac lymph nodes. She was initiated on salvage Romidepsin, Ifosfamide, Carboplatin, Etoposide (R-ICE) and completed cycle 1, 5 months following the diagnosis of AITL and 11 months following the diagnosis of ET.

### Patient 2

**Figure 3.**
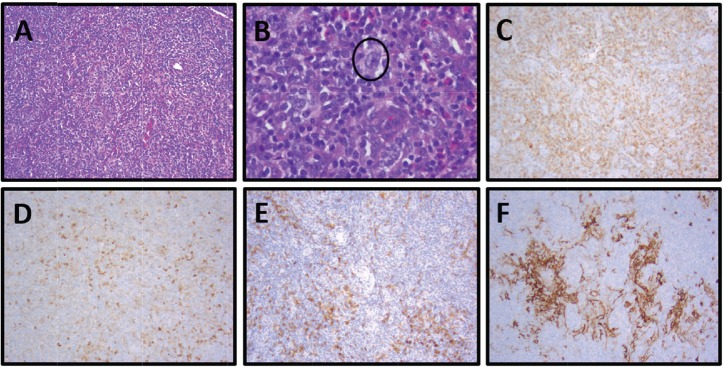
Patient 2, axillary lymph node with AITL: (A) Partial nodal architecture effacement by a monomorphic lymphoid population and increased numbers of eosinophils, H&E, 100×. (B) Neoplastic population of monomorphic follicular helper T cells with small to medium-sized nuclei and clear-to-pale cytoplasm in a polymorphous background of neutrophils and increased numbers of eosinophils with scattered CD30+ immunoblasts (circled, IHC not shown), H&E, 400x. (C) Increased numbers of neoplastic helper T cells, CD4, 100×. (D) Increased numbers of neoplastic follicular T helper cells (dim) with scattered neutrophils (strong), CD10, 100x. (E) Increased numbers of neoplastic helper T cells, PD-1, 100x. (F) Expanded dendritic meshwork, CD23, 100×.

An 84-year-old Caucasian man with a history of congestive heart failure and ET (*JAK2 V617F* mutation) diagnosed 6 years prior and treated with hydroxyurea presented for evaluation of peripheral blood eosinophilia. He noted weight loss and fatigue but denied night sweats or fevers. Physical examination was notable for palpable cervical, axillary and inguinal LAD, and a diffuse macular rash. Initial laboratory findings were notable for a peripheral eosinophilia of 17.7%, peripheral monocytosis of 19.4% and lymphopenia of 12.4%. CT of his chest, abdomen and pelvis noted diffuse LAD and splenomegaly. His bone marrow biopsy showed hypercellular marrow (50%) without evidence of malignancy. Corresponding flow cytometry indicated an increased CD4:CD8 ratio of 10:1 without aberrant T-cell antigen expression or a clonal B-cell population. His axillary lymph node biopsy with immunostaining was positive for CD2, CD3, CD4, CD5, CD7, CD10 (subsets), BCL-6 and PD-1 in the lesional T-cells, consistent with AITL (Figure 3; Table 1). EBV was negative by EBER. Cytogenetic analysis demonstrated a normal male karyotype (46, XY).

His PET/CT showed increased uptake in the right cervical, infraparotid and axillary lymph nodes, right upper lobe of the lung, and spleen. He was started on Cyclophosphamide, Etoposide, Vincristine and Prednisone (CEOP); adriamycin hydrochloride was held due to his heart failure. After three cycles, repeat PET/CT showed disease progression in all involved sites. The patient ultimately requested comfort/hospice care, 4 months after the diagnosis of AITL and 6 years after the diagnosis of ET.

### Patient 3

**Figure 4.**
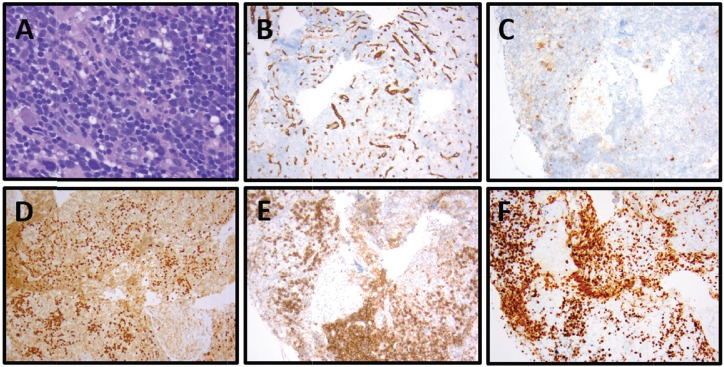
Patient 3, cervical lymph node with T-ALL. (A) Relatively monomorphic infiltrate of neoplastic lymphocytes with irregularly shaped, hyperchromatic, small to medium-sized nuclei with inconspicuous nucleoli, 400x. (B) CD34-negative, (CD34-positive blood vessels as internal control), 100x. (C) CD10-negative, 100x. (D) TdT-positive (nuclear), 100x. (E) CD1a-positive, 100x. (F) Ki-67 with 80% proliferation index, 100x.

A 62-year-old African American man with a history of PMF (*JAK2 V617F* mutation) presented 3 years after initial diagnosis with a 30-pound weight loss, night sweats and fatigue. Physical examination revealed bilateral posterior cervical LAD. The patient had a bone marrow biopsy that indicated fibrosis with 40% cellularity, but no evidence of malignancy. Cytogenetic analysis showed 46,XY,i(17)(q10) [9]/46,idem,del(20)(q11.2q13.1) [3]. His excisional cervical lymph node biopsy immunophenotype stained positive for Tdt, CD1a, CD2, CD7, cytoplasmic CD3, CD33 and CD34, and was negative for surface CD3 and MPO in the lesional T-cells, compatible with the diagnosis of T-ALL (Figure 4; Table 1). His PET/CT showed increased uptake in the left cervical and axillary lymph nodes. He was initiated on Cyclophosphamide, Vincristine, Doxorubicin and Dexamethasone (HyperCVAD), and, after six cycles, was in complete remission.

Two months later, he developed posterior cervical and supraclavicular LAD. His CBC was significant for pancytopenia, requiring transfusions of packed red blood cells. A repeat bone marrow biopsy indicated progressive marrow fibrosis and a focal area of TdT positivity. Bone marrow flow cytometry was negative for lymphoblastic cells. The patient had a CT scan of his chest, abdomen and pelvis, which demonstrated splenomegaly (18 cm) and diffuse LAD. A core biopsy of a left cervical lymph node was consistent with relapsed T-ALL (stage IV), so the patient was started on Nelarabine and Dexamethasone. After two cycles, the patient developed a diffuse papular rash. He had a punch biopsy that showed an atypical immature cell infiltrate within the dermis. Immunochemical staining was positive for CD45, MPO, CD43 and CD68, and negative for CD3, CD5, TdT, CD20, CD34, CD117 and CD1a in the lesional cells, consistent with the diagnosis of a cutaneous myeloid sarcoma. He was transitioned to Clofarabine and Cytarabine, which was complicated by neutropenic sepsis from *Escherichia Coli* bacteremia and acute appendicitis after cycle 1. Ultimately, the patient requested comfort/hospice care, 3 years after being diagnosed with PMF, and 1 year following the diagnosis of T-ALL.

## Discussion

These cases demonstrate the development of AITL and T-ALL in patients previously diagnosed with PN-MPN. This is the first case series of T-ALL or AITL arising in patients with PN-MPN, apart from one other reported case in the literature [11] (Table 1). While rarely described, these cases suggest a possible association between PN-MPN and T-cell malignancies. While the development of secondary lymphoid neoplasms remains rare, data suggest that the incidence is greater in males, in those with a *JAK2 V617F* mutation, and in patients with a pre-existing PN-MPN of greater than 5 years [12]. When including the other reported case with this case series, the median age was 70 years (range: 20–84 years) with a 3:1 male-to-female predominance, and a median duration of 4.5 years (range: 6 months–10 years) between the diagnosis of a PN-MPN and the development of a T-cell neoplasm (Table 1). All cases were advanced at diagnosis and exhibited rapid progression or relapse despite systemic chemotherapy and regardless of karyotype, MPN mutation and type of PN-MPN or T-cell neoplasm (Table 1).

The occurrence of a haematolymphoid malignancy following a previously diagnosed PN-MPN is uncommon. Nonetheless, clinicians should be aware that studies have demonstrated patients with PN-MPN are at increased risk of this complication. Vannucchi* et al* [12] reported a standardised incidence ratio (SIR) of 3.4 (95% confidence interval (95% CI): 1.9–6.2) and 12.4 (95% CI: 4.7–33.6) in patients who develop secondary chronic lymphocytic leukaemia and non-Hodgkin lymphoma compared with the general population, respectively. Furthermore, patients with PN-MPN harbouring the *JAK2 V617F* mutation had a SIR of 5.46 (95% CI: 2.45–12.15) of acquiring a secondary haematolymphoid malignancy [12]. In a Danish population-based cohort study, the SIR of developing a haematologic malignancy in patients with ET was 5 (95% CI: 3.5–6.9) [13]. The SIR in patients with ET was approximately equal to PV in the study and reported as 1.8 (95% CI: 1.1–2.7) and 0.6 (95% CI: 0.2–1.5) in patients with non-Hodgkin lymphoma and lymphoid leukaemia, respectively [13]. While it is well documented that haematolymphoid malignancies may lead to secondary myelofibrosis, PMF with a secondary haematolymphoid malignancy is rare and its occurrence is primarily isolated to case reports.

The pathophysiology by which a lymphoid transformation occurs in patients with PN-MPN arises has not been clearly established. However, mutations involving *JAK2 V617F* have been suggested as a potential contributor. There is evidence implicating lympho-myeloid progenitor cells as the cell of origin that culminates in PN-MPN [14]. The JAK-STAT pathway plays a fundamental role in lymphoid proliferation, survival and differentiation [15] and mutations have been observed in paediatric acute lymphoblastic leukaemias, adult T-cell lymphoblastic leukaemias and Hodgkin lymphoma [16, 17]. In addition, there are reported cases of the *JAK2 V617F* mutation in both PN-MPN as well as lymphoma cells following a lymphomatous transformation, suggestive of a causal link and mutational aetiology for the lymphomatous transformation [12]. Larsen *et al* [14] discovered a subpopulation of B and T lymphocytes with the *JAK2 V617F* mutation in a population of patients with PN-MPN, suggesting that this mutation occurs in an early stem cell with both myeloid and lymphoid differentiation potential. This may indicate that there exists a proportion of T-lymphocyte sub-clone precursors arising from a common early lympho-myeloid progenitor that harbour the mutation, creating a predisposition for eventual lymphomatous or leukaemic progression. An assessment of *JAK2* mutations was not done in the malignancies in these reported cases and given that the first patient had a triple-negative ET, there may be other mutations harboured by early progenitor cells that were not assessed.

Patient 1 developed AITL 6 months after the diagnosis of triple-negative ET. However, a triple-negative status does not exclude other less common genomic aberrancies [18] which may have also have contributed to the development of a secondary T-cell neoplasm. In 68 patients with previously diagnosed triple-negative ET, Zu *et al* [19] conducted targeted genomic sequencing and found other mutations in *FLT3*, *SH2B3*,* ASXL1*,* ADAMTS1*,* TET2*,* TP53*,* EGFR*,* CUX1*,* GATA2* and *MPL* that may have contributed to the development of ‘triple-negative’ ET. Several of these mutations are also seen in T-cell malignancies. In T-ALL, mutations in *FLT3*, *SH2B3* and *ASXL1* have been demonstrated [20, 21], and in AITL, mutations in *TET2*,* TP53* and *GATA2* (reported in EBV-associated T-cell lymphoma) have been reported [22–24]. These were not assessed in patient 1, but may have been contributing factors to disease pathogenesis.

## Conclusion

This case series demonstrates the possibility of developing T-cell neoplasms in patients with PN-MPN, independent of cytogenetic abnormalities and mutational status. Disease progression may be rapid, and disease recurrence may occur if remission is achieved. Clinicians managing patients with PN-MPN should be aware of this possibility and maintain a high index of suspicion for this potential complication when patients present with ‘B’ symptoms or new LAD. A detailed diagnostic workup should be conducted efficiently so that systemic therapy can be initiated expediently. Workup in T and B-cell malignancies that develop in patients with PN-MPN should include a mutation analysis of *JAK2 V617F*,* CALR* and *MPL.*

## Abbreviations

Myeloproliferative neoplasm: MPN; Philadelphia Negative Myeloproliferative Neoplasm: PN-MPN; WHO: World Health Organization; Polycythemia Vera: PV; Essential Thrombocythemia: ET; Primary Myelofibrosis: PMF; Janus Kinase: JAK; Calreticulin: CALR; Thrombopoietin Receptor: MPL.

## Conflicts of interest

The authors have no conflicts of interest.

## Funding statement

The authors have no financial disclosures to declare.

## Figures and Tables

**Table 1.** Characteristics of patients with MPN with secondary AITL and T-ALL.

	Patient 1	Patient 2	Patient 3	Aitchison et al [13]
Age(years)/Gender/ Ethnicity	78/Female/Caucasian	84/Male/Caucasian	62/Male/African American	20/Male/Unknown
MPN	ET	ET	PMF	PV
Mutation	Triple-Negative	JAK2 V617F	JAK2 V617F	Unknown
Treatment	Hydroxyurea	Hydroxyurea	Erythropoietin	Unknown
Time from MPN diagnosis to leukaemic transformation	6 months	6 years	3 years	10 years
Malignancy	AITL	AITL	T-ALL	T-ALL
Disease Stage at Diagnosis	IV	IV	IV	Unknown
Immunophenotype of lesional cells	CD2, CD3, CD5, CD7, CD4, PD-1	CD2, CD3, CD4, CD5, CD7, with subsets expressing CD10, BCL-6, and PD-1, CD30	Tdt, CD1a, CD2, CD7 and cytoplasmic CD3, CD33 and CD34	Unknown
Karyotype	47,XX,add(2)(p2),add(10)(p11.2),+add(20)(p12)[cp4]/46,XX [16].	46,XY	46,XY,i(17)(q10)[9]/46,idem,del(20)(q11.2q13.1) [3].	Unknown
Bone marrow infiltration	Yes	No	Yes	Unknown
EBV	Positive	Negative	Negative	Unknown
Chemotherapy	CHEOP, R-ICE	CEOP	HyperCVAD, Nelarabine, Clofarabine and Cytarabine	Unknown
Outcome	Disease progression, on salvage chemotherapy with R-ICE	Disease progression, deceased	Disease relapse within 2 months, cutaneous myeloid sarcoma, deceased	Deceased

MPN: Myeloproliferative neoplasm. ET: Essential Thrombocythemia; PV: Polycythemia Vera; PMF: Primary Myelofibrosis; AITL: Angioimmunoblastic Lymphoma; T-ALL: T-cell Acute Lymphoblastic Leukaemia; CHEOP: Cyclophosphamide, Doxorubicin hydrochloride, Etoposide, Vincristine and Prednisone; CEOP: Cyclophosphamide, Etoposide, Vincristine and Prednisone; R-ICE: Romidepsin, Ifosfamide, Carboplatin, Etoposide; HyperCVAD: Cyclophosphamide, Vincristine, Doxorubicin and Dexamethasone.
